# Celecoxib plus hormone therapy versus hormone therapy alone for hormone-sensitive prostate cancer: first results from the STAMPEDE multiarm, multistage, randomised controlled trial

**DOI:** 10.1016/S1470-2045(12)70088-8

**Published:** 2012-05

**Authors:** Nicholas D James, Matthew R Sydes, Malcolm D Mason, Noel W Clarke, John Anderson, David P Dearnaley, John Dwyer, Gordana Jovic, Alastair WS Ritchie, J Martin Russell, Karen Sanders, George N Thalmann, Gianfilippo Bertelli, Alison J Birtle, Joe M O'Sullivan, Andrew Protheroe, Denise Sheehan, Narayanan Srihari, Mahesh KB Parmar, for the STAMPEDE investigators

**Affiliations:** a School of Cancer Sciences, University of Birmingham, Birmingham, UK; b Medical Research Council Clinical Trials Unit, London, UK; c School of Medicine, Cardiff University, Cardiff, UK; d Christie and Salford Royal Hospitals Foundations Trusts, and Manchester University, Manchester, UK; e Royal Hallamshire Hospital, Sheffield, UK; f Institute of Cancer Research and Royal Marsden Hospital, Sutton, UK; g Prostate Cancer Support Federation, Stockport, UK; h Beatson West of Scotland Cancer Centre, Glasgow, UK; i Inselspital, Bern, Switzerland; j Singleton Hospital, Swansea, UK; k Royal Preston Hospital, Preston, UK; l University of Manchester, Manchester, UK; m Centre for Cancer Research and Cell Biology, Queen's University Belfast, Belfast, UK; n Churchill Hospital, Oxford, UK; o Royal Devon and Exeter, Exeter, UK; p Shrewsbury and Telford Hospital NHS Trust, Shrewsbury, UK

## Abstract

**Background:**

Long-term hormone therapy alone is standard care for metastatic or high-risk, non-metastatic prostate cancer. STAMPEDE—an international, open-label, randomised controlled trial—uses a novel multiarm, multistage design to assess whether the early additional use of one or two drugs (docetaxel, zoledronic acid, celecoxib, zoledronic acid and docetaxel, or zoledronic acid and celecoxib) improves survival in men starting first-line, long-term hormone therapy. Here, we report the preplanned, second intermediate analysis comparing hormone therapy plus celecoxib (arm D) with hormone therapy alone (control arm A).

**Methods:**

Eligible patients were men with newly diagnosed or rapidly relapsing prostate cancer who were starting long-term hormone therapy for the first time. Hormone therapy was given as standard care in all trial arms, with local radiotherapy encouraged for newly diagnosed patients without distant metastasis. Randomisation was done using minimisation with a random element across seven stratification factors. Patients randomly allocated to arm D received celecoxib 400 mg twice daily, given orally, until 1 year or disease progression (including prostate-specific antigen [PSA] failure). The intermediate outcome was failure-free survival (FFS) in three activity stages; the primary outcome was overall survival in a subsequent efficacy stage. Research arms were compared pairwise against the control arm on an intention-to-treat basis. Accrual of further patients was discontinued in any research arm showing safety concerns or insufficient evidence of activity (lack of benefit) compared with the control arm. The minimum targeted activity at the second intermediate activity stage was a hazard ratio (HR) of 0·92. This trial is registered with ClinicalTrials.gov, number NCT00268476, and with Current Controlled Trials, number ISRCTN78818544.

**Findings:**

2043 patients were enrolled in the trial from Oct 17, 2005, to Jan 31, 2011, of whom 584 were randomly allocated to receive hormone therapy alone (control group; arm A) and 291 to receive hormone therapy plus celecoxib (arm D). At the preplanned analysis of the second intermediate activity stage, with 305 FFS events (209 in arm A, 96 in arm D), there was no evidence of an advantage for hormone therapy plus celecoxib over hormone therapy alone: HR 0·98 (95% CI 0·90–1·06). 2-year FFS was 51% (95% CI 46–56) in arm A and 51% (95% CI 43–58) in arm D. There was no evidence of differences in the incidence of adverse events between groups (events of grade 3 or higher were noted at any time in 123 [23%, 95% CI 20–27] patients in arm A and 64 [25%, 19–30] in arm D). The most common grade 3–5 events adverse effects in both groups were endocrine disorders (55 [11%] of patients in arm A *vs* 19 [7%] in arm D) and musculoskeletal disorders (30 [6%] of patients in arm A *vs* 15 [6%] in arm D). The independent data monitoring committee recommended stopping accrual to both celecoxib-containing arms on grounds of lack of benefit and discontinuing celecoxib for patients currently on treatment, which was endorsed by the trial steering committee.

**Interpretation:**

Celecoxib 400 mg twice daily for up to 1 year is insufficiently active in patients starting hormone therapy for high-risk prostate cancer, and we do not recommend its use in this setting. Accrual continues seamlessly to the other research arms and follow-up of all arms will continue to assess effects on overall survival.

**Funding:**

Cancer Research UK, Pfizer, Novartis, Sanofi-Aventis, Medical Research Council (London, UK).

## Introduction

Prostate cancer is a major health problem worldwide, accounting for nearly a fifth of all newly diagnosed male cancers. In the UK, roughly 35 000 men are diagnosed with prostate cancer each year, and in 2008 almost 10 000 men died from the disease.^1^ Globally, 913 000 cases were diagnosed in 2008.^2^ The current standard first-line treatment for locally advanced or metastatic prostate cancer is hormone therapy, achieved either surgically with bilateral orchidectomy or medically with luteinising hormone releasing hormone (LHRH) agonists or antagonists, or oral antiandrogens,^3^ with additional radiotherapy for locally advanced cases.^4^, ^5^ Hormone therapy produces responses in up to 95% of patients, but it is not curative and disease recurs in nearly all patients; median time to progression is estimated as 18–24 months, driven by metastatic cases,^3^ and is longer in patients with locally advanced disease.^4^, ^5^ Such disease is referred to as hormone-refractory prostate cancer (HRPC), or increasingly as castrate-refractory prostate cancer (CRPC), although androgen-deprivation-refractory prostate cancer might be a preferable term. In that setting, there is now a range of systemic treatments, including further hormonal manipulations,^6^ bisphosphonates,^7^ cytotoxic chemotherapy,^8^ radionuclides,^9^ immunotherapy,^10^ and newer hormone therapies.^11^ The traditional approach is to assess new treatments for prostate cancer in castrate-refractory disease. An alternative approach is to investigate new drugs and new approaches to treatment as first-line therapy in patients starting hormone therapy. At this point, patients are potentially fitter and better able to tolerate treatment, and intervention in the hormone-naive setting might have a better and more durable effect.

The STAMPEDE trial (Systemic Therapy for Advanced or Metastatic Prostate cancer: Evaluation of Drug Efficacy; Medical Research Council [MRC] PR08) is an innovative, multiarm, multistage (MAMS), multicentre, randomised controlled trial. We designed the trial to assess the effects of a bisphosphonate (zoledronic acid), a cytotoxic chemotherapy drug (docetaxel), and a cyclo-oxygenase-2 (COX-2) inhibitor (celecoxib), as single agents or combinations, in patients starting hormone therapy for locally advanced or metastatic prostate cancer. The trial is designed with separate stages focusing on safety, activity, and efficacy data; a research arm is only allowed to proceed to the final stage of recruitment if the study treatment is shown to be acceptably safe and sufficiently active. We refer to lack of sufficient activity as lack of benefit.

COX-2 is an isoenzyme induced by various mitogens, cytokines, and growth factors that are associated with a range of processes including inflammation^12^ and carcinogenesis.^13^, ^14^ Various case-control studies have shown a reduction in risk of prostate cancer associated with the use of non-steroidal anti-inflammatory drugs (NSAIDs), which include inhibition of COX-2 among their mode of action.^15^ Pathological studies show that COX-2 is upregulated in carcinomas,^16^ and one study suggested that NSAID use might delay progression from subclinical to clinical prostate cancer.^17^ This combination of preclinical and epidemiological data justified assessment of a COX-2 inhibitor in the present trial.

The trial development group (including clinical researchers, statisticians, trialists, and patient representatives) chose to assess the selective COX-2 inhibitor, celecoxib, because data suggest that it is better tolerated than other NSAIDs, exhibits activity as a cancer-preventing agent,^18^ and shows inhibition of angiogenesis and induction of apoptosis in human cancer cells including prostate cancer,^19^ particularly at higher doses.^20^ This decision was taken after full consideration of the risks and benefits, in view of reports of potential adverse cardiovascular effects.^21^

No safety concerns were raised during the pilot phase and the first intermediate activity analysis, and the celecoxib arms were permitted to continue accrual. Here, we report the results of the second intermediate analysis comparing hormone therapy alone with hormone therapy plus celecoxib.

## Methods

### Study design and participants

STAMPEDE uses an adaptive multiarm, multistage design.^22^, ^23^ This seamless phase 2–3 design starts with several trial arms and uses an intermediate outcome to adaptively focus accrual away from the less encouraging research arms, continuing accrual only with the more active interventions. The definitive primary outcome of the STAMPEDE trial is overall survival. The intermediate primary outcome is failure-free survival (FFS) defined as the first of: PSA failure (PSA >4 ng/mL and PSA >50% above nadir); local progression; nodal progression; progression of existing metastases or development of new metastases; or death from prostate cancer. FFS is used as a screening method for activity on the assumption that any treatment that shows an advantage in overall survival will probably show an advantage in FFS beforehand, and that a survival advantage is unlikely if an advantage in FFS is not seen. Therefore, FFS can be used to triage treatments that are unlikely to be of sufficient benefit. It is not assumed that FFS is a surrogate for overall survival; an advantage in FFS might not necessarily translate into a survival advantage.

The trial is managed by a trial management group (TMG) chaired by the chief investigator. Accumulating comparative data are reviewed by the independent data monitoring committee (IDMC) and recommendations are made to the trial steering committee (TSC), which includes independent members, who have the final responsibility for decision making (eg, on stopping arms). The TSC can view limited accumulating comparative trial data to take appropriate action.

Patients were recruited from specialist centres with the appropriate local and national approvals. The eligibility criteria (detailed in the appendix) encompassed a range of patients requiring treatment with long-term hormone therapy. We postulated that the relative effect of the research treatments would be the same across trial arms, even if the absolute event rate differed. We included patients with newly diagnosed prostate cancer with metastases to bone, node-positive disease, or clinically localised disease with high-risk features (at least two of the following: T category 3 or 4, Gleason sum score 8–10, or PSA ≥40 ng/mL). Additionally, men failing previous localised therapy were eligible (subject to restrictions on prior hormone therapy) if they had a PSA concentration of 4 ng/mL or higher and doubling time of less than 6 months, or PSA 20 ng/mL or higher, or nodal or metastatic relapse.

Patients had to be fit for any of the trial treatments, with adequate haematological, renal, and liver function, and have a WHO performance status of 0 or 1. Patients with a confirmed history of severe cardiovascular problems, active peptic ulceration, gastrointestinal bleeding, or inflammatory bowel disease were excluded. All patients provided written informed consent.

### Randomisation and masking

Computer-based randomisation was done centrally (via telephone) using minimisation with a random element of 80% allocation towards minimising arms, balancing on minimisation factors of randomising centre, metastases, nodal involvement, age at randomisation, WHO performance status, type of hormone therapy, regular aspirin or NSAID use at baseline, and planned use of radiotherapy. Patients could be allocated to any of the trial arms. This trial is open label; masking was considered impracticable because of the requirement for intravenous delivery of zoledronic acid and docetaxel. Further details on the design and conduct of the trial are presented elsewhere.^24^, ^25^

### Procedures

All patients were planned to receive long-term hormone therapy for at least 2 years, and were allowed to start long-term hormone therapy up to 12 weeks before randomisation. The permitted methods of hormone therapy were LHRH analogues (with short-term antiandrogens to cover disease flare when necessary), maximum androgen blockade, LHRH antagonists, orchidectomy, or, in patients without metastasis, bicalutamide monotherapy (150 mg daily); choice of hormone therapy was based on clinician and patient preference. Radiotherapy was encouraged for men with N0M0 disease (stratifying on intent), with a target window of 6–9 months after starting hormone therapy, so that radiotherapy could be given at the same time in all trial arms and patients would not have chemotherapy and radiotherapy concomitantly. Patients allocated to celecoxib were planned to receive one 400 mg capsule twice daily, taken orally, until 1 year or an FFS event.

Patients were followed up every 6 weeks for 6 months, then every 12 weeks for 2 years, then every 6 months for 5 years, and annually thereafter. PSA measurements were done at every follow-up; further tests were at the discretion of the treating clinician. Nadir PSA was considered the lowest value within 6 months on trial. Toxicities and symptoms were systematically reported at each follow-up; serious adverse events and reactions were reported according to the National Cancer Institute Common Toxicity Criteria.

### Statistical analysis

The sample size was calculated using -nstage- and its predecessor programs. This program, which is implemented in Stata, is freely available^23^ and allows for the design of multiarm, multistage trials. We assumed, for the control arm, median FFS of 2 years and median overall survival of between 4 and 5 years, depending on patient mix. We targeted a 25% relative reduction in events (hazard ratio [HR] 0·75) for both FFS and survival; this translates to a 9% absolute improvement at the median time. Table 1 shows the trial design parameters, which start with a permissive alpha and become stricter over time. These parameters were chosen to ensure that meaningful amounts of new data would be accumulated between intermediate analyses, which were triggered when specific numbers of events had been reported in the control group (table 1). A one-sided test was chosen because research arms must pass an intermediate hurdle to continue accrual. The power was set high throughout the stages to avoid excluding an active research arm inappropriately. The overall alpha and power levels represent the values for each pairwise comparison of research arm against the common control arm, accounting for intermediate analyses and repeated use of the control arm.

**Table 1:** Statistical design parameters according to study stage

	**Primary outcome**	**Hazard ratio**	**Power**	**One-sided alpha**	**Critical hazard ratio**	**Control events**
Stage I: activity	FFS	0·75	95%	0·500	1·00	114
Stage II: activity	FFS	0·75	95%	0·250	0·92	216
Stage III: activity	FFS	0·75	95%	0·100	0·89	334
Stage IV: efficacy	OS	0·75	90%	0·025	..	405

FFS=failure-free survival. OS=overall survival.

The statistical design parameters translate into a series of activity hurdles against which each research arm is compared at three predefined intermediate analyses.^24^ At the end of the second activity stage, reported here, the HR cutpoint was 0·924, determined with 95% power and a one-sided alpha of 0·25, with analyses planned for when roughly 216 FFS events had been reported in the control group. It was anticipated that research arms with an HR less favourable than the cutpoint would be considered as showing insufficient activity and accrual might therefore be stopped; accrual would continue to the remaining trial arms to collect further evidence.

There is no single sample size target for STAMPEDE; the trial targets a total of 405 deaths in the control arm in comparisons with active research arms, but the number of patients required to observe these events depends on the accrual rate, actual event rate (mix of patients joining the trial), and number of research arms shown to be insufficiently active at the intermediate stages. The duration of accrual and follow-up are adjusted to obtain this target.^24^ We anticipated that around 2500–4000 patients would join STAMPEDE over 6–8 years, with around 1500 patients in comparisons of research arms showing encouraging evidence at each intermediate analysis. Twice as many patients are recruited to the control group as to any of the research groups. This is because the control group is in every pairwise comparison and the power of these comparisons is improved by having more patients in this group.

Standard survival analysis methods were used for time-to-event data. A log-rank test was used to compare event times using the Kaplan-Meier method. Cox's proportional hazards models were used to estimate the relative treatment effects, adjusting for key stratification factors, with relative improvements expressed as HRs; HR less than 1·00 favours a research arm. The proportional hazards assumption was tested; provision was made to report restricted mean survival times and to translate the stopping boundaries appropriately in the instance of a non-proportional treatment effect over time.^26^ All CIs are provided at the 95% level for tradition, but a one-sided 75% CI is also included for primary comparison of FFS, in line with the trial design. All patients are included in the analyses on an intention-to-treat basis.

Toxicities and symptoms were considered together from routinely collected toxicity reports and spontaneously reported serious adverse event (SAE) datasets. Patients who had not returned follow-up (or SAE) data were conservatively excluded from the safety dataset and assessment of toxicity. The proportions of patients with grade 3–5 toxicities were not formally compared. Analyses were done with Stata (version 11).

The trial is registered with ClinicalTrials.gov, number NCT00268476, and with Current Controlled Trials, number ISRCTN78818544.

### Role of the funding source

The trial was sponsored by the MRC and conducted by the MRC Clinical Trials Unit. MRC employees were central to the conduct of the trial and the development of this manuscript. Only authors MRS and GJ had access to raw data; processed data released by the IDMC and TSC were available to all coauthors. Cancer Research UK approved the trial design but had no further input. Pfizer, Novartis, and Sanofi-Aventis approved the trial design and participated in discussions on the progress of the trial. Representatives from these industry partners were invited to comment on the manuscript but only typographic issues were noted. The analyses were driven by prespecified criteria and the decision to submit for publication was made by the TMG, following guidance from the IDMC and TSC. The corresponding author had full access to all of the data and the final responsibility to submit for publication.

## Results

**Figure 1:**
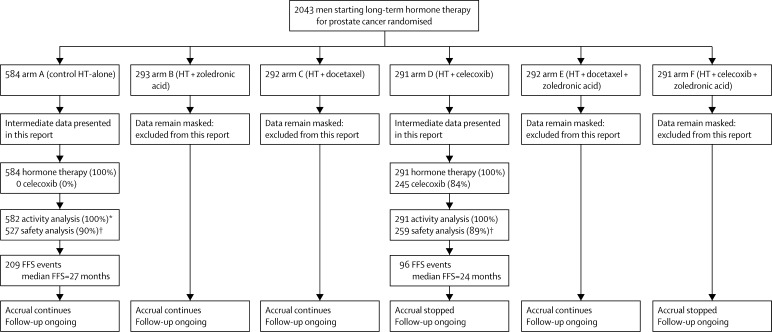
Trial profile HT=hormone therapy. FFS=failure-free survival. *Two patients were excluded from the activity analysis in arm A because of errors in event dates that were unresolved at the time of this intermediate analysis. Both patients reported FFS events before randomisation. †Patients are conservatively excluded from the safety analysis if they have not returned follow-up data or reported a serious adverse event; this is expected since accrual was ongoing at the time of analysis and some patients would not have reached their first on-trial assessment point.

Data were frozen on Feb 1, 2011, for review by the IDMC on March 31, 2011. Between Oct 17, 2005, and Jan 31, 2011, STAMPEDE recruited 2043 patients from 85 centres in the UK and Switzerland. 584 patients were randomly allocated to receive hormone therapy alone and 291 to receive hormone therapy plus celecoxib. Figure 1 shows the flow of patients through the trial; table 2 gives the baseline characteristics of patients in this comparison.

**Table 2:** Baseline characteristics

	**HT only (n=584)**	**HT + celecoxib (n=291)**
**WHO performance status**		
0	448 (77%)	223 (77%)
1	127 (22%)	64 (22%)
2	8 (1%)	4 (1%)
Missing	1	0
**Age (years)**		
Median (IQR)	65 (59–70)	65 (60–71)
Range	41–82	41–80
Missing	0	0
**PSA (ng/mL)**		
Median (IQR)	67 (24–201)	58 (23–175)
Range	1–15 747	2–5000
Missing	0	0
**Days from diagnosis**		
Median (IQR)	75 (51–99)	70 (52–96)
Range	0–3594	1–3359
Missing	45	30
**Pain from prostate cancer**		
Absent	428 (81%)	232 (89%)
Present	99 (19%)	29 (11%)
Missing	57	30
**T category**		
T0	4 (1%)	1 (0%)
T1	10 (2%)	6 (2%)
T2	52 (9%)	16 (5%)
T3	376 (64%)	191 (66%)
T4	97 (17%)	52 (18%)
TX	45 (8%)	25 (9%)
**N stage**		
N0	266 (46%)	132 (45%)
N+	278 (48%)	138 (47%)
NX	40 (7%)	21 (7%)
**Metastases**		
None	229 (39%)	115 (40%)
One or more sites	355 (61%)	176 (60%)
Bone metastasis	308 (53%)	151 (52%)
Liver metastasis	8 (1%)	2 (<1%)
Lung metastasis	18 (3%)	5 (2%)
Nodal metastasis	109 (19%)	49 (17%)
Other metastasis	23 (4%)	19 (7%)
**Aspirin or NSAID use**		
No	436 (75%)	218 (75%)
Yes	148 (25%)	73 (25%)
**Planned or current HT**		
LHRH	574 (98%)	286 (98%)
Orchidectomy	3 (<1%)	2 (<1%)
Bicalutamide	7 (1%)	3 (1%)
Missing	39	26
**Time from starting HT (days)**		
Median (IQR)	38 (15–60)	32 (16–53)
Range	−34 to 105	−31 to 97
Missing	6	3
**Planned antiandrogen use**		
Short-term antiandrogen	387 (84%)	183 (82%)
Long-term antiandrogen	74 (16%)	41 (18%)
Missing	123	67
**Radiotherapy planned**		
No	439 (75%)	220 (76%)
Yes	145 (25%)	71 (24%)
**Participation in QoL study**		
No	4 (2%)	1 (<1%)
Yes	208 (98%)	103 (99%)
Not invited*	372	187
**Smoking status**		
No	460 (86%)	237 (87%)
Yes	77 (14%)	35 (13%)
Missing on cardiovascular assessment	7	3
Cardiovascular assessment not yet received	40	16
**Diabetes**		
No	494 (91%)	251 (91%)
Yes, type 1	11 (2%)	6 (2%)
Yes, type 2	38 (7%)	18 (7%)
Missing on cardiovascular assessment	1	0
Cardiovascular assessment not yet received	40	16
**Myocardial infarction**		
No	531 (98%)	268 (97%)
Yes, but still fit for trial	11 (2%)	7 (3%)
Missing on cardiovascular assessment	2	0
Cardiovascular assessment not yet received	40	16
**Cerebrovascular disease**		
No	538 (99%)	272 (99%)
Yes, but still fit for trial	4 (1%)	3 (1%)
Missing on cardiovascular assessment	2	0
Cardiovascular assessment not yet received	40	16
**Congestive heart failure**		
No	538 (100%)	273 (99%)
Yes, but still fit for trial	2 (<1%)	2 (1%)
Missing on cardiovascular assessment	4	0
Cardiovascular assessment not yet received	40	16
**Angina**		
No	525 (97%)	264 (96%)
Yes, but still fit for trial	17 (3%)	10 (4%)
Missing on cardiovascular assessment	2	1
Cardiovascular assessment not yet received	40	16
**Hypertension**		
No	355 (65%)	179 (65%)
Yes, but still fit for trial	188 (35%)	96 (35%)
Missing on cardiovascular assessment	1	0
Cardiovascular assessment not yet received	40	16

HT=hormone therapy. PSA=prostate-specific antigen. NSAID=non-steroidal anti-inflammatory drug. LHRH=luteinising hormone releasing hormone. QoL=quality of life.

* After Oct 21, 2008, the QoL substudy stopped recruiting new patients; around 700 patients were in the trial at that point.

**Figure 2:**
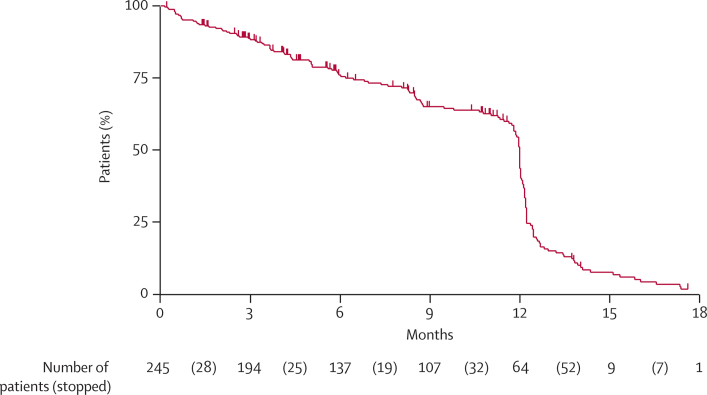
Time on celecoxib for patients in arm D (hormone therapy plus celecoxib) Only patients who reported starting celecoxib treatment are included. Patients still on celecoxib are censored at the date of last treatment. Patients were provided with sufficient tablets for 12 months of treatment. The drop at 12 months reflects patients completing their trial treatment; events before then represent patients stopping trial treatment sooner, for any reason.

In both arms, androgen-deprivation therapy was LHRH based in nearly all patients (574 of 584 [98%] in the control group and 286 of 291 [98%] in the hormone therapy plus celecoxib group), with radiotherapy planned for around a quarter of patients. More than one-third of patients stopped celecoxib sooner than 1 year after randomisation (figure 2). The reason for stopping celecoxib was known for 107 of these patients: 45 (42%) completed protocol treatment, 30 (28%) had disease progression, 12 (11%) had excessive toxicity, and 20 (19%) stopped for other reasons. Data on radiotherapy are not available at this stage.

209 FFS events were observed in the control group and 96 in the hormone therapy plus celecoxib group. Median FFS was 27 months (95% CI 20–39) in the control group, with an FFS at 2 years of 51% (95% CI 46–56). In the hormone therapy plus celecoxib group, median FFS was 24 months (95% CI 17–33), with an FFS at 2 years of 51% (95% CI 43–58).

In the 209 patients in the control group who had an FFS event, the first reported event was PSA failure in 163 patients (78%), metastases in 33 (16%), local progression in five (2%), lymph-node progression in five (2%), and prostate-cancer-related death in three (1%). In the 96 patients in the hormone therapy plus celecoxib group who reported an FFS event, the first event was PSA failure in 75 (78%), metastases in 15 (16%), local progression in two (2%) and prostate-cancer-related death in four (4%).

**Figure 3:**
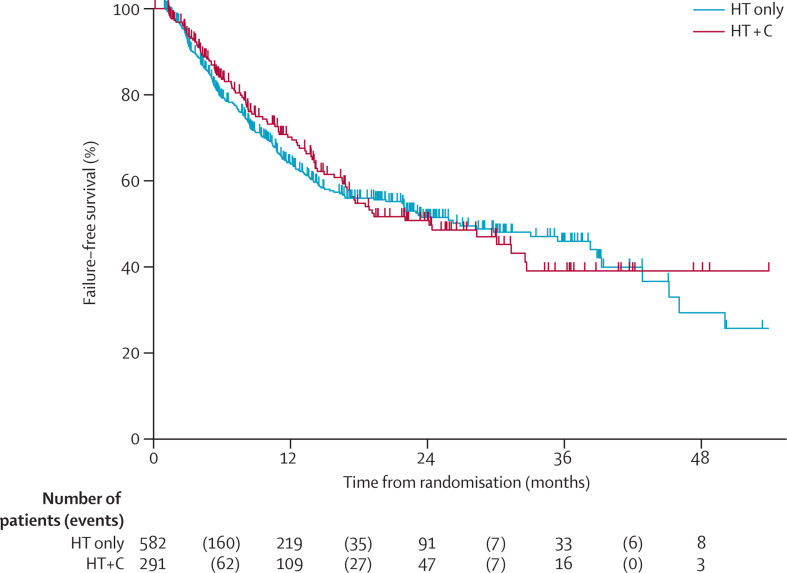
Kaplan-Meier curve of failure-free survival in arm A (hormone therapy alone) versus arm D (hormone therapy plus celecoxib) HT=hormone therapy. C=celecoxib.

At this second intermediate analysis, celecoxib showed insufficient evidence of activity, in terms of FFS, for continuation of accrual to this comparison: HR 0·98 from adjusted Cox model (75% CI upper limit 1·01; 95% CI 0·90–1·06; figure 3). The HR point estimate was less favourable than the prespecified activity cutpoint of 0·924. Because of the lack of benefit indicated by this analysis, the IDMC recommended discontinuing recruitment to both celecoxib-containing groups (hormone therapy plus celecoxib [arm D], and hormone therapy plus celecoxib plus zoledronic acid [arm F]), and this was endorsed by the TSC. Treatment with celecoxib was also discontinued in both arms. Treatment with zoledronic acid continues in the combination group (arm F).

There is some evidence of non-proportional hazards in the data, which is mostly explained by WHO performance status (p=0·007); there was no evidence that the hazards for the treatment effect are non-proportional over time (p=0·387). A flexible parametric model with three degrees of freedom allowing for time-varying treatment effects estimated an HR of 0·96 (95% CI 0·88–1·05). Other sensitivity analyses were consistent with the lack of additional activity of celecoxib (data not shown). The restricted mean FFS time over the first 48 months on trial was 26·9 months in the hormone therapy plus celecoxib group compared with 26·7 months in the control group; the difference was 0·3 months (95% CI −5·3 to 5·8; p=0·92).

The safety dataset includes 786 patients (90%): 527 in the hormone therapy alone group and 259 in the hormone therapy plus celecoxib group. Grade 3–5 toxicities were reported at any time by 123 (23%; 95% CI 20–27) patients in the control group and 64 (25%, 19–30) in the hormone therapy plus celecoxib group. Grade 3–5 events were mainly endocrine effects related to hormone therapy in both groups (table 3). There was no evidence of treatment-related cardiovascular dysfunction.

**Table 3:** Incidence of adverse effects any time after randomisation, by category

	Hormone therapy alone (n=527)*			**Hormone therapy plus celecoxib (n=259)**		
	Grade 1–2	Grade 3–4	Grade 5	Grade 1–2	Grade 3–4	Grade 5
Cardiac disorder	42 (8%)	7 (1%)	1 (<1%)†	37 (14%)	5 (2%)	0 (0%)
Renal	99 (19%)	9 (2%)	0 (0%)	53 (20%)	2 (<1%)	1 (<1%)
Endocrine disorder	363 (70%)	55 (11%)	0 (0%)	162 (63%)	19 (7%)	0 (0%)
Musculoskeletal	322 (61%)	30 (6%)	0 (0%)	135 (52%)	15 (6%)	0 (0%)
General disorder	301 (58%)	15 (3%)	0 (0%)	137 (53%)	8 (3%)	0 (0%)
Nervous system	118 (23%)	9 (2%)	0 (0%)	52 (20%)	6 (2%)	0 (0%)
Respiratory	113 (22%)	7 (1%)	0 (0%)	64 (25%)	7 (3%)	0 (0%)
Gastrointestinal disorder	238 (46%)	8 (2%)	0 (0%)	131 (51%)	5 (2%)	0 (0%)
Laboratory abnormalities	117 (23%)	9 (2%)	0 (0%)	62 (24%)	4 (2%)	0 (0%)
Hepatic disorder	45 (9%)	8 (2%)	0 (0%)	20 (8%)	5 (2%)	0 (0%)
Skin	118 (23%)	5 (<1%)	0 (0%)	62 (24%)	2 (<1%)	0 (0%)
Blood or bone marrow	24 (5%)	3 (<1%)	0 (0%)	9 (3%)	2 (<1%)	0 (0%)
Blood and lymphatic	89 (17%)	3 (<1%)	0 (0%)	47 (18%)	1 (<1%)	0 (0%)
Psychiatric	143 (28%)	3 (<1%)	0 (0%)	72 (28%)	0 (0%)	0 (0%)
Metabolic and nutritional	53 (10%)	1 (<1%)	0 (0%)	23 (9%)	2 (<1%)	0 (0%)
Allergic reaction	19 (4%)	1 (<1%)	0 (0%)	11 (4%)	1 (<1%)	0 (0%)
Ocular	39 (8%)	1 (<1%)	0 (0%)	16 (6%)	0 (0%)	0 (0%)
Peripheral oedema	64 (12%)	0 (0%)	0 (0%)	31 (12%)	0 (0%)	0 (0%)

* Percentages account for missing data for some of the toxicities for between two and nine patients.

† Complication of cardiac intervention.

Activity and safety data for all other trial arms were reviewed by the IDMC but were not released for further review. These data, including those for the hormone therapy plus celecoxib plus zoledronic acid group, remain confidential to the IDMC and will only be available to the TSC after future analyses.

## Discussion

In the STAMPEDE trial, we are assessing several drugs in combination with hormone therapy in patients with high-risk localised or metastatic prostate cancer. Resources are focused on trial arms most likely to show a clinically meaningful survival benefit by using intermediate lack-of-benefit analyses and stopping accrual to arms showing insufficient activity or adverse safety profiles.

The celecoxib arm continued accrual through the first intermediate analysis (target HR 1·00), but accrual was stopped after the second intermediate analysis when the target HR was more difficult to pass, at 0·92. Recruitment to the hormone therapy plus celecoxib group was stopped with immediate effect. Although there were no safety concerns raised by the IDMC, there was no evidence of benefit when the totality of the data were taken into consideration, and the TSC recommended that treatment with celecoxib stop for patients still receiving the drug.

Our premise is that an advantage in overall survival should be preceded by an advantage in FFS, so we do not anticipate a later benefit with celecoxib emerging; however, this remains to be determined in subsequent analysis of overall survival after longer follow-up. In some CRPC studies this assumption has not held up, particularly studies with the immunotherapy sipuleucel-T, where a survival advantage has been shown without a prior benefit in PSA progression.^10^ The potential mechanisms of action of celecoxib are not androgen-linked, so it is reasonable to assume that they might not be reflected in PSA-based measures of FFS; on this basis, we continue long-term patient follow-up and avoid definitive statements relating to the overall efficacy of COX-2 inhibition and prostate-cancer survival in this setting.

Recruitment was also stopped to the hormone therapy plus celecoxib plus zoledronic acid group; data for this arm have not been revealed to avoid inappropriate influence on recruitment in the other zoledronic acid-containing arms. These data will subsequently become available, giving further information from an additional 450 patients randomised to receive celecoxib as part of treatment. The patients in both celecoxib-containing groups remain in the trial and will continue to be followed up to provide data on overall survival. Subgroup analyses by disease stage were done but not made available for reporting. The IDMC charter allows it to make recommendations within a subgroup if warranted by the data. There would have been limited power for such a comparison at this stage.

Celecoxib, a selective COX-2 inhibitor, was selected on the basis of preclinical^13^, ^14^, ^19^ and clinical^16^, ^17^, ^18^ data suggesting possible utility in prostate cancer (panel). The celecoxib dose and treatment duration chosen for the trial (400 mg twice daily for 12 months) was based on the dose used for prevention of familial polyposis coli^20^ and the need to minimise potential for cardiovascular risk, which seems to present with treatment durations longer than 12 months.^21^ The dose and duration were selected after a comprehensive literature review followed by joint discussion with patient representatives on the TMG, emphasising the importance of such collaboration in the design and conduct of clinical trials.

Panel **Research in context**

**Systematic review**

At the time of trial design, there was substantial epidemiological, laboratory, and clinical evidence that COX-2 has a role in development and progression of a range of cancers, including prostate cancer. Celecoxib was chosen for assessment in hormone-sensitive prostate cancer based on in-vitro evidence of activity and data in other cancers, particularly familial polyposis coli, where it has a role in prevention of progression from polyp to cancer. We assessed a range of drugs with COX-2-inhibitory properties and selected celecoxib based on the data in familial polyposis coli and its safety profile in large-scale trials of other diseases. Data from trials of celecoxib in established cancers have been tracked through the registers (including alerts and ClinicalTrials.gov), and lead investigators have been contacted for information each time reviews are updated but registers do not include recent data.

**Interpretation**

At the second preplanned intermediate analysis, we have shown that celecoxib given at 400 mg twice daily for 1 year is insufficiently active in high-risk, hormone-sensitive prostate cancer to significantly affect failure-free survival. Other trials in prostate cancer (and other cancer types) have not supported the use of COX-2 inhibitors unequivocally in any setting, and our trial adds further evidence of the limited clinical utility of these drugs in established, advanced cancer. We do not recommend their use in these patients.

External clinical trial data that have emerged since the launch of STAMPEDE have been equivocal as to the value of COX-2 inhibitors in prostate^27^, ^28^ and colorectal^29^, ^30^ cancers. So far, published trials have not supported the use of COX-2 inhibitors unequivocally in any established cancer type; our trial adds further evidence of their limited clinical utility in established tumours. We do note positive findings for chemoprevention of non-melanoma skin cancer in patients with actinic keratoses.^31^ Several trials of celecoxib in other types of established tumours continue to recruit patients, including large trials in colon (eg, CALGB-80702 [NCT01150045]), bladder (eg, BOXIT [ISRCTN84681538]), and breast cancer (eg, REACT [ISRCTN48254013]).

We cannot identify at this stage why a drug with a sound pretrial rationale would show no evidence of activity in a large-scale trial, but there are several possibilities to consider. First, there could be lack of expression of the target molecule COX-2, which we could retrospectively assess by collecting tissue blocks and studying COX-2 expression. Second, there might be a lack of on-target activity; celecoxib was chosen because of documented activity in the setting of familial polyposis coli,^20^ thus it seems reasonable to assume that the drug dose and delivery are within the necessary therapeutic range. Third, the dose or duration of exposure of celecoxib, or both, may have been inadequate. The initial planned duration of therapy was 2 years, but in view of the excess cardiovascular problems reported with rofecoxib^29^ just before accrual to STAMPEDE, a shorter duration was selected. Even if the duration was too short for optimum effect, we would still expect some effect, particularly with a median time to progression of around 2 years.

A key strength of STAMPEDE is that several therapeutic combinations are tested synchronously, thereby shortening the time to assess efficacy and toxicity in new drug combinations in hormone-naive patients undergoing hormone therapy. A further strength is that recruitment is broadly based, incorporating more than 100 centres in two countries. The entry criteria were intended to define a population with a poor prognosis, requiring long-term hormone therapy, and to test the hypothesis that interventions at the point of first hormone manipulation improve long-term outcome. The trial is predicated on the notion that high-risk prostate cancer includes a range of patients, from those with localised disease and poor prognostic characteristics (based on T category, PSA, and Gleason score) to those presenting with metastatic disease, and that current therapies have limited long-term efficacy in many cases. The original statistical design therefore assumed a median FFS time of around 2 years; this estimation has been validated by the data presented here. Median overall survival was assumed to be twice the median FFS, although there have been too few deaths to estimate this accurately. It seems likely that median survival will be higher than originally anticipated, which might be partly related to new, active therapies that patients can receive after their first trial FFS event. These therapies include docetaxel,^32^, ^33^ which has entered standard practice for treatment of later stages of prostate cancer since the trial was launched. Further agents are emerging, including abiraterone,^34^ cabazitaxel,^35^ sipuleucel-T,^10^ radium-223,^36^ and MDV3100.^37^, ^38^ With the recent demonstration of a survival benefit with radical radiotherapy in patients with locally advanced disease,^4^, ^5^ we anticipate further improvements in FFS and overall survival; indeed, the trial was amended in November, 2011, to mandate the use of radiotherapy in newly diagnosed N0M0 disease following these results.^5^ The accumulating event rate is monitored as part of trial oversight processes.

The remaining trial arms continue to recruit new patients and their data remain masked. Researchers might infer that there is at least some evidence of improved FFS in these arms, but such evidence is not sufficient to stop or change the trial. A further change to the trial occurred in November, 2011, with the introduction of hormone therapy plus abiraterone as a new research arm. Patients in the new arm will only be compared with those randomised contemporaneously to the control group. The trial can adaptively introduce further research arms and stop early those arms showing a lack of benefit.^24^ Further research arms are likely to be introduced into the trial. The practical issues in handling the early stopping of the celecoxib-containing arms and the addition of further research arms will be discussed in a separate paper. In conclusion, we have shown that celecoxib given 400 mg twice daily for 1 year is insufficiently active for men starting long-term therapy for high-risk prostate cancer, and we do not recommend its use in this setting.
