# Supplementary material for: Celecoxib plus hormone therapy versus hormone therapy alone for hormone-sensitive prostate cancer: first results from the STAMPEDE multiarm, multistage, randomised controlled trial
Source: Lancet Oncol. 2012 May;13(5):549–58. doi: 10.1016/S1470-2045(12)70088-8 (PMC3398767; doi:10.1016/S1470-2045(12)70088-8)
Supplement: Supplementary appendix [file mmc1.pdf]

THE LANCET Oncology

Supplementary appendix

This appendix formed part of the original submission and has been peer reviewed. We post it as supplied by the authors.

Supplement to: James ND, Sydes MR, Mason MD, et al, for the STAMPEDE investigators. Celecoxib plus hormone therapy versus hormone therapy alone for hormone-sensitive prostate cancer: first results from the STAMPEDE multiarm, multistage, randomised controlled trial. *Lancet Oncol* 2012; published online March 26. DOI:10.1016/S1470-2045(12)70088-8.

Supplemental Table 1: Eligibility criteria by protocol version

	Date and Version					
	**V1.1**	**V2**	**V3**	**V4**	**V5**	**V6**
**Eligibility criteria**	**May-05**	**Jun-05**	**Jul-06**	**Dec-07**	**Aug-08**	**Jul-09**
**Inclusion criteria - disease**						
Patients must fulfil at least one of the criteria [A] or [B]	X	X	X	X	X	X
[A] High Risk Newly Diagnosed Patients with one of:-						
→ Stage T3/4 N0 M0 histologically confirmed prostate adenocarcinoma with PSA>=40ng/ml or Gleason sum score 8-10	X	X	X	-	-	-
→ Fulfil at least two out of the three following criteria: Stage T3/4 N0 M0 histologically confirmed prostate adenocarcinoma, PSA>=40ng/ml or Gleason sum score 8-10	-	-	-	X	X	X
→ Stage T_{any} N+ M0 or T_{any} N_{any} M+ histologically confirmed prostate adenocarcinoma	X	X	X	X	X	X
→ Multiple sclerotic bone metastases with a PSA>=100ng/ml without histological confirmation	X	X	X	X	X	X
[B] Patients with histologically confirmed prostate adenocarcinoma previously treated with radical surgery or radiotherapy who are now relapsing with at least one of:-						
→ PSA >=4ng/ml and rising with doubling time less than 6 months	X	X	X	X	X	X
→ PSA >=20ng/ml	X	X	X	X	X	X
→ N+	-	-	-	-	X	X
→ M+	-	-	-	-	X	X
**Inclusion criteria – other issues**						
Intention to treat with long-term hormone therapy	X	X	X	X	X	X
Fit for all protocol treatment and follow-up, WHO performance status 0-2	X	X	X	X	X	X
Have completed the appropriate investigations prior to randomisation	X	X	X	X	X	X
Adequate haematological function: neutrophil count >1.5X10^9/l and platelets >100X10^9/l	X	X	X	X	X	X
Adequate renal function: Serum creatinine <1.5 ULN	X	X	X	X	X	X
Adequate liver function: ALT or AST <1.5 ULN, bilirubin <ULN	X	X	X	X	X	X
Normal testosterone level prior to hormone treatment	X	X	-	-	-	-
Written informed consent	X	X	X	X	X	X
Willing and expected to comply with follow-up schedule	X	X	X	X	X	X
**Exclusion criteria**						
Prior systemic therapy for locally advanced or metastatic prostate cancer except as listed in footnote	X	X	X	X	X	X
Metastatic brain disease or leptomeningeal disease	X	X	X	X	X	X
Any other previous or current malignant disease which, in the judgement of the responsible physician, is likely to interfere with STAMPEDE treatment or assessment	X	X	X	X	X	X
Patients with active peptic ulceration, gastrointestinal bleeding, inflammatory bowel disease	-	-	X	X	X	X
Symptomatic peripheral neuropathy grade 2 (NCI CTC)	X	X	X	X	X	X
Any surgery (e.g. TURP) performed within the past 4 weeks	X	X	X	X	X	X
Renal insufficiency with estimated creatinine clearance <30ml/min	X	X	X	X	X	X
Patients who have been on a Cox-2-inhibitor for at least 6 months prior to trial entry	X	X	X	X	X	X

	Date and Version					
	**V1.1**	**V2**	**V3**	**V4**	**V5**	**V6**
**Eligibility criteria**	**May-05**	**Jun-05**	**Jul-06**	**Dec-07**	**Aug-08**	**Jul-09**
Patients with confirmed cardiovascular history e.g.:<br>a. Severe/unstable angina<br>b. Myocardial infarction<br>c. Severe cardiac failure (NYHA II-IV*)<br>d. Cerebrovascular disease (eg stroke or transient ischaemic episode)	X	X	X	X	X	-
Patients with confirmed severe cardiovascular history e.g.:<br>a. Severe/unstable angina<br>b. Myocardial infarction<br>c. Severe cardiac failure (NYHA II-IV*)<br>d. Cerebrovascular disease (eg stroke or transient ischaemic episode)	-	-	-	-	-	X
Patients who have scheduled to have major dental extractions within the next 2 years	-	-	X	X	X	X

Supplemental Table 2: Participating sites

Participating site	Principle Investigator	Patients
**United Kingdom**		
Glasgow, Beatson West of Scotland Cancer Centre	Martin Russell	148
Cardiff, Velindre Hospital	Malcolm Mason	141
Birmingham, Queen Elizabeth Hospital	Nicholas James	97
Manchester, Christie Hopsital	Noel Clarke	78
Oxford, Churchill Hospital	Andrew Protheroe	75
Exeter, Royal Devon and Exeter Hospital	Denise Sheehan	69
Sutton-London, Royal Marsden Hospital	David Dearnaley	53
Preston, Royal Preston Hospital	Alison Birtle	53
Swansea, Singleton Hospital	Gianfilippo Bertelli	52
Belfast, City Hospital	Joe O’Sullivan	51
Shrewsbury, Royal Shrewsbury Hospital	Narayanan Srihari	50
Edinburgh, West General Hospital	Duncan McLaren	48
Southend, University Hospital	David Tsang	48
Torbay, Torbay District General Hospital,	Anna Lydon	44
Sheffield, Weston Park Hospital	Catherine Ferguson	42
Hull, Cottingham, Castle Hill Hospital	John Hetherington	40
Guildford, Royal Surrey Hospital	Robert Laing	37
Middlesbrough, James Cook University Hospital	John Hardman	37
London, Guy’s Hospital	Simon Chowdhury	35
Romford Queen's Hospital	Stephanie Gibbs	35
Kent, Maidstone Hospital	Sharon Beesley	34
Nottingham, University Hospital City Campus	Santhanam Sundar	34
Stockport, Stepping Hill Hosp	John Logue	33
Brighton, Royal Sussex County Hospital	Angus Robinson	30
Bournemouth, Royal Bournemouth Hospital	Sue Brock	29
Derby, London Road Community Hospital	Prabir Chakraborti	29
Northwood, Mount Vernon Hospital	Peter Hoskin	29
Warrington, Warrington Hospital	Isabel Syndikus	29
Burton on-Trent, Queen's Hospital	Prabir Chakraborti	29
Huddersfield, Huddersfield Royal Infirmary	Uschi Hofmann	29
Worthing, Worthing Hospital	Ashok Nikapota	27
Taunton, Musgrove Park Hospital	John Graham	27
Blackburn East Lancashire Trust	Omi Parikh	27
Sunderland, Sunderland Royal Hospital	Ian Pedley	27
Manchester, Hope Hospital	Noel Clarke	27
Wirral, Clatterbridge Centre for Oncology	Helen Innes	22
Bristol, Haematology and Oncology Centre	Amit Bahl	22
Dudley, Russells Hall Hospital	Prakash Ramachandra	19
Ipswich, Ipswich Hospital	Robert Brierly	16
Hereford, Hereford County Hospital	Audrey Cook	16
Southampton, Southampton General Hospital	Catherine Heath	15
High Wycombe, Wycombe Hospital	Andrew Protheroe	15
Inverness, Raigmore Hospital	Neil McPhail	14
Eastbourne, Eastbourne District General Hospital	Fiona McKinna	13
Harlow, Princess Alexandra Hospital	Nishi Gupta	12
Crewe, Leighton Hospital	James Wylie	11
Portsmouth, Queen Alexandra Hospital	Joanna Gale	11
Reading, Royal Berks Hospital	Paul Rogers	10

Participating site	Principle Investigator	Patients
Worcester, Worcestershire Royal Hospital	Jo Bowen	10
Durham, University Hospital	Rhona McMenemin	9
Chelmsford, Broomfield Hospital	Priscilla Leone	9
Leeds, St James University Hospital	William Cross	9
London, University College Hospital	Stephen Harland	9
Poole, Poole Hospital	Sue Brock	9
Ayr, Ayr Hospital	Jawaher Ansari	9
Swindon, Great Western Hospital	David J Cole	8
Liverpool, Royal Liverpool University Hospital	Zafar Malik	8
Basingstoke, Basingstoke and North Hampshire Hospital	Richard Shaffer	7
London, North Middlesex Hospital	Jackie Newby	7
Southport, Southport and Formby District General Hospital	Asha Sivapalasuntharam	7
Bath, Royal United Hospital	Hugh Newman	7
Stevenage, Lister Hospital	Robert Hughes	7
Keighley Airedale Hospital	Michael Crawford	6
Kent, Kent and Canterbury Hospital	Natasha Mithal	6
Manchester, Withington Community Hospital	Vijay Sangar	5
St Leonards-on-Sea, Conquest Hospital	Sharon Beesley	5
London, St George’s Hospital	Lisa Pickering	5
London, Royal Free Hospital	Maria Vilarino-Varela	5
Darlington, Darlington Memorial Hospital	John Hardman	4
Chester Countess of Chester Hospital	Azman Ibrahim	4
Aylesbury, Stoke Mandeville Hospital	Andrew Protheroe	3
Bolton, Royal Bolton Hospital	Tony Elliott	3
Wigan, Royal Albert Edward Infirmary	Richard Cowan	3
Dorset County Hospital	Perric Crellin	3
Suffolk, West Suffolk Hospital	Cathryn Woodward	2
Ashford, William Harvey Hospital	Natasha Mithal	1
Bradford Royal Infirmary	Simon Brown	1
Devon, North Devon District Hospital	Denise Sheehan	1
London, St Mary's Hospital	Alison Falconer	1
Sutton-in-Ashford, King's Mill Hospital	Daniel Saunders	1
**Switzerland**		
Chur Switzerland, Kantonsspital Graubunden	Raeti Strebel	4
St Gallen Switzerland, Kantonsspital St Gallen	Daniel Engeler	3
Berne Switzerland, Inselpital - University Hospital	George Thalmann	1
Zurich Switzerland, Triemlispital	D. Siciliano	1
Aarau Switzerland, Hirslanden Medical Centre	Razvan Popescu	1
